# Assessment of bone mineral density by DXA and the trabecular microarchitecture of the calcaneum by texture analysis in pre- and postmenopausal women in the evaluation of osteoporosis

**DOI:** 10.4103/0971-6203.37481

**Published:** 2007

**Authors:** R. Karunanithi, S. Ganesan, T. M. R. Panicker, M. Paul Korath, K. Jagadeesan

**Affiliations:** KJ Hospital Research and Postgraduate Centre, Chennai, Tamil Nadu, India; * Division of Medical Physics, Department of Physics, Anna University, Chennai, Tamil Nadu, India

**Keywords:** Bone mineral density, osteoporosis, texture analysis, trabecular architecture

## Abstract

The *in vivo* evaluation of trabecular bone structure could be useful in the diagnosis of osteoporosis for the characterization of therapeutic response and understanding the role of parameters other than bone mineral density (BMD) in defining skeletal status. This study was made to evaluate changes taking place in the trabecular architecture of bone with age and menopausal status in women. The findings are compared with the femoral neck bone as well as the trochantar bone mineral density determined by dual energy X-ray absorptiometry (DXA), which is a standard reference test for evaluation of osteoporosis. Seventy females were recruited for the study, 25 premenopausal (mean age ± SD: 39.4 ± 3.8) and 45 postmenopausal (mean age ± SD: 57.9 ± 7.9) women. The right femoral neck bone mineral density was measured for them by dual energy X-ray absorptiometry (DXA). For the same individuals, lateral view radiographs of the right calcaneum were taken as well. The radiographs were digitized and the region of interest (ROI) of 256 × 256 pixels was selected, the run length matrix was computed for calculating seven parameters [Table 1] and the two dimensional fast Fourier transform of the image was calculated. Using the FFT, the power spectral density (PSD) was derived and the root mean square (RMS) value was determined. Our results confirm that age has a significant influence on the texture of the trabecular bone and bone mineral density.

Osteoporosis has been defined as ‘a disease characterized by low bone mass and microarchitectural deterioration of bone tissue, leading to enhanced bone fragility and a consequent increase in fracture risk’ (WHO-1994). Data about prevalence of this disease worldwide shows that 25% of women over the age of 50 succumb to breakage of bone due to low bone mass, and half of them (12.5%) have risk of osteoporosis. In case of men, about 8% of them have been found to suffer from osteoporosis. In India the published data was scarce till 1990, though there were several cases of bone fracture and low bone mass. Now as per estimation, there are about 12 million cases of osteoporosis and further increments are likely due to greater longevity, poor calcium and vitamin D intake, nutritional fads and poor acceptability of hormone replacement therapy (HRT).

Bone mineral density is known to decrease as age advances. Osteoporosis causes significant morbidity and loss of quality of life. Mortality is greater in patients who have osteoporosis in middle-aged and older populations.[1] Especially, the condition is more frequent in postmenopausal women. Osteoporosis is characterized by an abnormal loss of bone mineral content, which leads to a tendency toward nontraumatic bone fractures or to structural deformations of bone.[2] Accurate estimation of the bone mineral density (BMD) has been an important diagnostic indicator for determining osteoporosis and for follow-up study of the patient under the therapy for osteoporosis. In this context, various BMD-measuring tools have been developed. Dual energy X-ray absorptiometry (DXA) and quantitative computed tomography (QCT) are typical methods of measuring BMD.

Though BMD is a useful concept, it does not give information about the trabecular structure of the bone. Noninvasive and/or nondestructive techniques can provide structural information about bone, beyond standard bone mineral density (BMD). While the latter provides important information about osteoporosis diagnosis and fracture risk assessment, considerable evidence indicates that BMD only partially explains bone strength and fracture resistance. Quantitative assessment of macro-structural characteristics such as geometry and section modulus; and micro-structural features such as relative trabecular volume and trabecular spacing, number and connectivity may improve our understanding of osteoporosis and our ability to estimate bone strength and predict fractures. The rationale for imaging bone macro-structure/microstructure, therefore, is to obtain information beyond BMD, improve fracture risk prediction, clarify the pathophysiology of skeletal disease, define the skeletal response to therapy and assess biomechanical relationships.

The most important aspect of osteoporosis is fractures in femoral neck and vertebrae. Especially, fracture in femur leads to about 20% mortality in case of older osteoporotic populations. Though BMD is a major indicator of bone strength, many studies have also shown that BMD alone cannot fully predict the possibility of osteoporotic fracture[3] and that other factors such as microstructure of trabecular bone and loading distribution have a significant effect on osteoporotic bone fracture.[3] In addition to the BMD measure, it is necessary to monitor the corresponding alterations in the trabecular microarchitecture.

Medical image processing has become the most important research topic, with development of various imaging tools and high-performance computer facilities. In particular, plain radiographic image processing has been extensively studied because radiography is widely available and relatively inexpensive. One of the well-known diseases which can be screened via plain radiographic images is osteoporosis.

Osteoporosis is established when decrease in bone mass greater than that expected for a person of a given age, sex and race is present and when it results in structural bone failure manifested by the occurrence of fractures following trivial trauma. Therefore, microstructure of trabecular bone, in addition to bone mineral density, should be considered to better predict the possibility of osteoporotic fractures. In the past decade, structural measures of trabecular bones have been studied in relation with osteoporotic fracture risk. Three-dimensional measure of trabecular bone may be the ideal measure. Recently, flat-panel-based micro CT has been extensively studied and will be used for real three-dimensional studies of trabecular bone. On the other hand, many studies have been focused on quantifying two-dimensional trabecular pattern in slice images of CT and magnetic resonance (MR). Studies on plain radiographs have been also performed to assess *in vivo* trabecular structures. These studies are mainly done on anatomic sites such as femur and the spine.[4,6] The calcaneum has been chosen as the site of measurement on the skeleton as it is rich in trabecular bone and is not covered by thick soft tissue. Trabecular structure of the bone is dynamic and contributes to the strength of the bone. Bone mineral density gives a value of the average distribution of bone mineral at the site of interest, i.e., the areal density. However, bone mineral in reality is distributed three-dimensionally in the trabecular architecture whose number and orientation contribute to bone strength. Therefore, study of the trabecular structure of bone is likely to be of great value in evaluating osteoporosis, in addition to bone mineral density.

## Materials and Methods

### Material

All the subjects voluntarily entered the study, after receiving information and giving informed consent by signing the relevant form. The Institutional ethics committee had approved and cleared the study protocol. We excluded patients with other conditions likely to interfere with bone integrity; and patients with malignancy, endocrine diseases (affecting thyroid, parathyroid and adrenal glands), Paget's disease, long-term immobilization, chronic renal failure or rheumatoid arthritis. Also, patients with diabetes, hyperthyroidism, bone cancers; fractures by severe trauma were excluded from the study.

Twenty-five pre-menopausal (mean age ± SD: 39.4 ± 3.8) and 45 postmenopausal women (mean age ± SD: 57.9 ± 7.9) participated in the study. (One woman had fracture in the spine at the thoracolumbar vertebra, and another had a hip fracture in the femur neck.)

### Methods

#### Working principles of DXA:

When a three-dimensional absorber (such as human body) is scanned by X-ray, it produces two-dimensional flat image on the photographic film. The human body does not act as a homogeneous absorber; a single energy X-ray beam cannot differentiate among the different components such as fat mass, lean mass and bone. For this, dual energy X-ray technique was utilized. Bone mineral density measurement using dual energy X-ray absorptiometry (DXA) has great clinical significance in the early detection and diagnosis of osteoporosis. X-ray absorption is the basic mechanism for discrimination between organs in a body under X-ray observation (Aston, 1990). Exactly how much X-ray is absorbed by different tissues is determined by Lambert's law and is given by

I=Ioe−μx

where I is the X-ray intensity emerging from tissue, I_o_ is the X-ray intensity incident on the tissue, x is the tissue thickness and μ is the mass attenuation coefficient.

**Figure 1:**
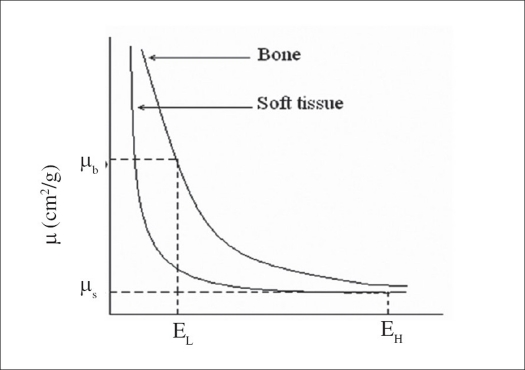
The mass attenuation coefficient versus X-ray photon energy

The DXA principle is based on the fact that mass attenuation coefficient (μ_m_) for different tissues decreases at different rates with increase in X-ray energy. At low X-ray energy, mass attenuation coefficient of bone (μ_b_) is very high compared to soft tissue (μ_s_); and at high X-ray energy, ‘μ_b_’ is approximately equal to that of ‘μ_s_’ as shown in Figure 1.

#### BMD measurements by dual energy X-ray absorptiometry (DXA):

In the clinical setting, the hip and spine should be the site for BMD measurement; because hip fractures are associated with higher morbidity and mortality, and vertebral compression fractures in the thoracic and lumbar spine are the most frequent clinical manifestations of osteoporosis. In our study, the Bone Mineral Density of the right hip was measured by DXA (Hologic QDR – 4500 densitometer). In the proximal femur bone, mineral densities from different sites were measured, including femoral neck, intertrochanteric area, trochanter and Ward's triangle. Total femoral BMD was also calculated as an average value of bone mineral density for the femur sites.

#### Calculation of T-score:

Osteoporosis is diagnosed by measuring bone mineral density (BMD), thereby defining thresholds. This is possible due to the Gaussian distribution of bone density values, where bone density is expressed in relation to a reference population in terms of standard deviation (SD) units (Kanis JA, 2002). When SD units are used in relation to a young healthy population, the measurement is referred to as the T-score [Table 1]. The T-score is the parameter that compares subject's BMD with average peak BMD of young normal population of the same gender.

**Table 1:** Assessment of bone mineral density by dual energy X-ray absorptiometry in osteoporosis (diagnostic categories expressed as T-scores)

Normal bone density	Patient BMD is greater than 1 SD below young adult reference mean BMD (T-score > −1).
Osteopenia	Patient BMD is between 1 SD and 2.5 SD below young adult reference mean BMD (T-score < −1 and > −2.5).
Osteoporosis	Patient BMD is 2.5 SD or more below young adult reference mean BMD (T-score < 2.5).
Severe osteoporosis	Patient BMD is 2.5 SD or more below young adult reference mean BMD with fragility fractures.

T-score=(Subject's BMD−Mean Young normal BMD)Standard deviation of Young normal BMD

Bone mineral density is defined as bone mineral content divided by the projected area of the scanned image.

BMD = BMC/area (g/cm^2^)

In our study group, the standard analysis procedure for the hip was performed on all the subjects as recommended by the manufacturer. The two measurements - namely, the femoral neck BMD (FN-BMD) and trochantar BMD (TR-BMD) - at the proximal femur were taken for the correlation with the texture parameters. The effective dose to the patient was 0.01 m Sev per DXA scan.

All the subjects underwent DXA tests and were classified as normal (*N* = 40), osteopenia (*N* = 22) and osteoporotic (*N* = 8). The mean and standard deviations of age of the individuals in the three groups were 46.5 years ± 9, 55.7 years ± 11 and 63.1 years ± 9 respectively. One case in the female group (age 73) had a nontraumatic compression and wedge type fracture in the thoracolumbar spine, noted elsewhere on a radiograph 1 year ago. Another lady (age 62) had fracture of the left femoral neck, treated elsewhere 1 year ago. Both had medication with calcium supplements since the occurrence of fracture. Patients with history of fracture due to severe trauma (such as patients met with accidents) were excluded from the study.

#### Radiography and digitization procedure:

**Figure 2:**
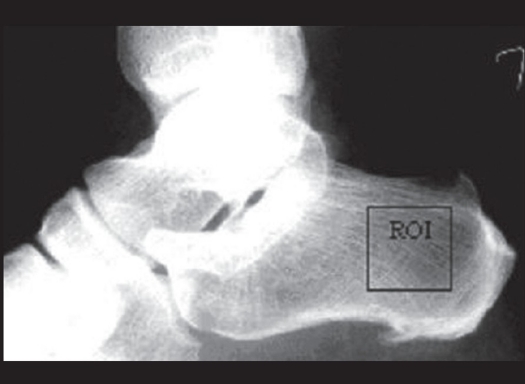
The region of Interest (ROI) cropped at posterior compressive trabecular network

Radiograph of the calcaneum lateral view were taken for all participants, according to a standardized protocol. The X-ray tube, at a setting of 46 kV and 4 mAs (100 mA and 0.04 s), was focused on the calcaneum; and the film-tube distance was fixed to 90 cm. Patient X-ray exposure to radiation using standard X-ray equipment was 1 μSev per examination.[8] The radiographs were digitized with a Kodak Film Digitizer (12 bits per pixel) and were stored as Tagged Image File Format (TIFF) files for analysis. The regions of interest (ROIs) were selected at the clinically significant area as shown in the diagram [Figure 2] and fed into the algorithm developed by us in Matlab software.

#### Acquisition of radiograph images for analysis:

The minimum intensity was subtracted from the cropped region of interest (ROI). The ROI was normalized (i.e., the gray level varies from 0 (intensity-value assigned for black pixel) to 255 (intensity value assigned for white pixel). Hence the gray level histogram of all the ROIs follows a normal or Gaussian distribution. These image-preprocessing techniques are carried out to make all the images uniform and also to ensure the reduction in the signal-to-noise ratio in the computation procedure.

#### Run-length matrix:

The gray level run length (GLRL) method is a way of extracting higher-order statistical texture features. The technique has been described and applied by Galloway (1975).[9–10] A set of consecutive pixels with the same gray level, collinear in a given direction, constitutes a gray level run. The run length is the number of pixels in run, and the run length value is the number of times such a run occurs in an image.

The gray level run length matrix (GLRLM) is a two-dimensional matrix in which each element p (i, j | θ) gives the total number of occurrences of runs of length ‘j’ at gray level ‘i’, in a given direction θ.

A number of scalar texture features may be computed from GLRLM - short runs emphasis (SRE), long runs emphasis (LRE), gray level non-uniformity (GLN), run-length non-uniformity (RLN), run percentage (RP), low gray level run emphasis (LGRE) and high gray level run emphasis (HGRE) [Table 2]

**Table 2:** Texture parameters and the formulae derived from gray level run length matrix

*Run-length matrix parameters*	*Formulae*
Short runs emphasis (SRE)	1/S S r (j | θ)/j^2^
Long runs emphasis (LRE)	1/S S r (j | θ) j^2^
Gray level non-uniformity (GLN)	1/S S g (i | θ)^2^
Run-length non-uniformity (RLN)	1/S S r (j | θ)^2^
Run percentage (RP)	1/n S r (j | θ)
Low gray level run emphasis (LGRE)	1/S S g (i | θ)/i^2^
High gray level run emphasis (HGRE)	1/S S i2 g (i | θ)

p (i, j | θ) is the (i, j)^th^ element of the run length matrix for a direction θ, G is the number of gray levels, R is the longest run, n is the number of pixels in the image, S is the total number of runs in the image.

#### Root mean square (RMS):

A fast Fourier transform was then performed, and the resulting power spectrum was analyzed to yield the root mean square (RMS) value.[2]

RMS=∑|F(u, v)|2/M*N

where RMS is the root mean square, |F(u,v)|^2^ is the power spectral density derived from the fast Fourier transform, and M*N is the size of the region of interest (ROI).

The trabecular bone pattern in the calcaneal radiographs was analyzed by a computerized texture analysis method that has been developed by us in Matlab 6.1. The digitized radiographs were converted to a pixel depth of 8 bits/pixel, i.e., 255 gray levels. Regions of interest (ROIs), 256 × 256 pixels in size (pixel size 100 μm), were then manually cropped at the posterior compressive trabecular bodies [Figure 1] and fed into the algorithm that computes the run-length matrix parameters and root mean square (RMS).

#### Reproducibility:

The precision of the selection of the region of interest from the same radiograph was estimated by repeatedly cropping the ROI of 256 × 256 pixels twenty five times at different intervals. The coefficients of variation for the texture parameters were 0.85%, 1.02%, 0.76%, 0.84%, 0.97% for five parameters (those mean values are found to be significant among pre- and postmenopausal women), which are all derived from run-length parameters; and was 1.08% for RMS.

The radiograph of the calcaneum was repeated for the same individual two times, and the region of interest from each digitized radiograph was cropped and the coefficient of variation was calculated and it was found to be 2.7%.

#### Data analysis:

**Figure 3:**
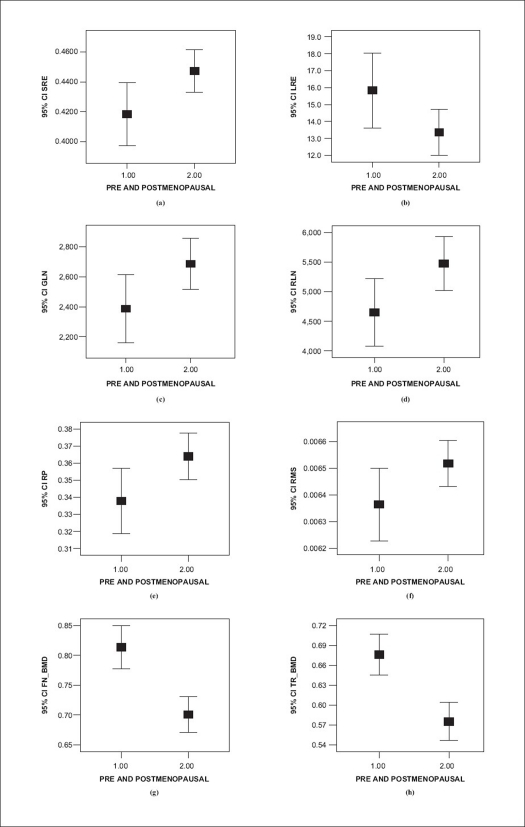
a-e (run-length parameters) and (f) RMS at significant 95% confident interval, (g) femoral neck BMD and (h) trochantar BMD in pre- and postmenopausal women

**Figure 4:**
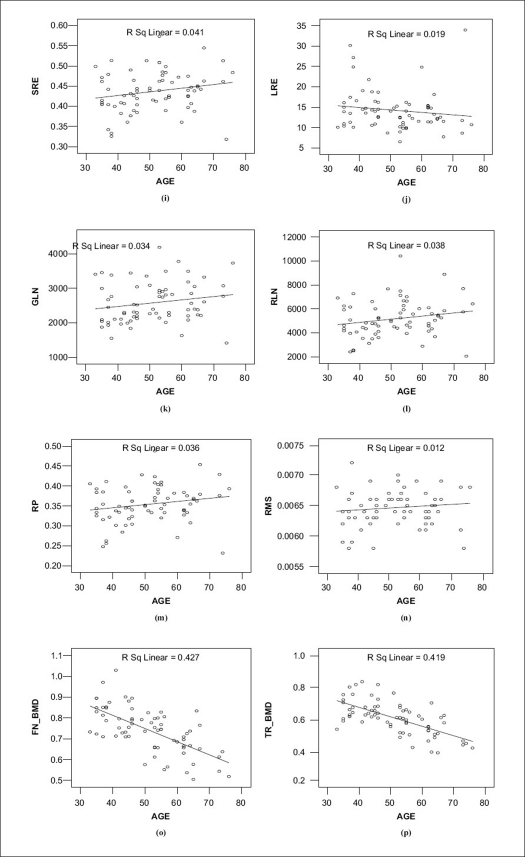
i-m (run-length parameters) and (n) RMS at significant 95% confident interval, (o) femoral neck BMD and (p) trochantar BMD in pre- and postmenopausal women

The mean values of the FN-BMD, TR-BMD and the mean values of the run-length matrix derived parameters and the mean RMS value of the PSD were compared using Student's *t*-test for pre- and postmenopausal women. A visual representation of these values by error bars with 95% confident intervals is shown in Figure 3, and the linear regressions are shown in Figure 4

The age has an influence on bone density and on trabecular bone texture analysis.[10] There is a notable decrease in the bone mineral density and an increase in RMS values in advanced age, as is evident from the graph.

## Results

The values of mean and standard deviation of age and number of pre-menopausal and postmenopausal women falling under the groups ‘normal,’ ‘osteopenia’ and ‘osteoporosis’ based on the T-score are tabulated [Table 3](classifications based on the local reference data[11]). The mean and SD of run-length-derived parameters and the RMS values for pre-menopausal women and postmenopausal women are tabulated in Table 4

**Table 3:** Subjects classified as ‘normal,’ ‘osteopenia’ and osteoporosis

Groups*	*Age (Mean ± SD)*	*Pre- menopause*	*Post- menopause*	*Total*
Normal	46.5 ± 8.9	19	21	40
Osteopenia	55.7 ± 11	6	16	22
Osteoporosis	63.1 ± 8.7	0	8	8
Total		25	45	70

SD is standard deviation.

* Grouped based on T-score [Femoral neck bone mineral density measured by dual energy X-ray absorptiometry (DXA)]

**Table 4:** Mean and standard deviation of the signifi cant texture values and bone mineral density values among the pre- and postmenopausal women

*Criteria*	*Age (Mean ± SD)*	*SRE (Mean ± SD)*	*LRE (Mean ± SD)*	*GLN (Mean ± SD)*	*RLN (Mean ± SD)*	*RP (Mean ± SD)*	*RMS (Mean ± SD)*	*FN_BMD (Mean ± SD)*	*T R _ B M D (Mean ± SD)*
Pre-menopause (N = 25)	39.4 ± 3.8	0.418 ± 0.05	15.83 ± 5.37	2387.75 ± 554.34	4649.92 ± 1370.6	0.337 ± 0.04	0.00636 ± 0.00006	0.813 ± 0.087	0.676 ± 0.075
Postmenopause (N = 45)	57.9 ± 7.9	0.447 ± 04	13.35 ± 4.48	2687.68 ± 567.7	5473.028 ± 1514.7	0.363 ± 045	0.00651 ± 0.00004	0.700 ± 0.098	0.575 ± 0.095
Significance	*P* = 0.000	*P* = 0.020	*P* = 0.043	*P* = 0.036	*P* = 0.028	*P* = 0.025	*P* = 0.046	*P* = 0.000	*P* = 0.000
r-value	-	0.041	0.019	0.034	0.038	0.036	0.012	0.427	0.419

The mean FN-BMD was 0.813 ± 0.087 g/cm^2^ for pre-menopausal women and 0.700 ± 0.098 for postmenopausal women. The mean TR-BMD was 0.676 ± 0.075 g/cm^2^ for pre-menopausal women and 0.575 ± 0.095 g/cm^2^ for postmenopausal women [Table 4]. The significant level and the confidence interval of the differences for all the texture parameters are given in the Table 5.

**Table 5:** Statistical analysis of the Independent Samples t-Test for two groups (pre and postmenopausal women)

*Texture parameter*	*Sig. (2-tailed)*	*95% confidence interval of the difference*	
		
		Lower	Upper
SRE	0.020^a^	−0.0531358	−0.0046278
LRE	0.043^a^	0.0817635	4.8774570
RLN	0.028^a^	−1552.5500	−93.651100
RP	0.025^a^	−0.0487566	−0.0034190
GLN	0.036^a^	−580.19290	−19.663900
LGRE	0.728^b^	−0.0019623	0.0027952
HGRE	0.718^b^	−6.6517999	9.6047635
RMS	0.046^a^	−0.0003040	−0.0000029

a *P* < 0.05 (significant);

b Not significant.

## Discussion

These results shows that the run-length matrix derived parameters and the RMS [derived from the power spectral density measure (PSD)] can predict significant alterations in the trabecular architecture, which may be comparable with the decrease in the FN-BMD and TR-BMD as age advances. Other groups have worked on nonfractal characterization of texture. The shape of the trabecular pattern on radius radiographs[12] showed that this shape correlated with lumbar BMD and age.

Several attributes were studied in parallel by one group on the radiographic bone images.[13] The statistical methods expressed some local relations between the gray levels of the image; the structural methods studied the distribution and shape of the radiographic patterns of the trabecular bone; and fractal analysis studied the roughness of the image texture by analyzing self-similarity variations over different scales.

In textural characterization, other attempts to evaluate the trabecular structure noninvasively have been undertaken. High-definition macro-radiography and fractal signature were used to analyze and to quantify the trabecular organization in vertebrae,[14] and they characterized architectural differences between groups of patients with low and high BMD. The fractal dimension was a better discriminator than lumbar spine BMD for distinguishing spine fracture cases.[15] The same type of analysis on proximal femur radiographs was done and showed that the fractal dimension correlated with compressive strength.[16] In a group of 10 cases of osteoporosis (bone density below normal and/or vertebral fracture) and a group of 10 controls, radius radiographs by a fractal method based on the Fourier transform were performed and showed that the mean fractal dimension of the two groups was significantly different (*P* < 0.05).[17]

## Conclusions

The texture parameters derived by the run-length matrix and the power spectral density (PSD) of bone texture on calcaneus radiographs constitute a new, simple, low-radiation and reproducible assessment of bone status. In this study we have shown that five out of seven texture parameters could predict changes in trabecular network. In addition to *in-vivo* BMD measure, the alterations in the trabecular network due to transition from pre-menopausal stage to postmenopausal stage were also quantified by the texture analysis of the radiographs. Hence both the BMD and micro-architecture undergo changes with respect to advancing age. This noninvasive analysis may provide information about the trabecular microarchitecture that is independent of bone density but depends on age. This method could be complementary to BMD measurements in assessing bone fragility. Adding information about the trabecular architecture to the bone mineral density would better predict osteoporosis where the DXA-derived bone mineral density had limitations in discriminating the osteoporotic fractured subjects from osteoporotic controls.

Out of seven texture measures derived from run-length matrix, five parameters and the RMS derived from PSD are found to be statistically significant *P*<0.05 among pre and postmenopausal women showing the evidence of notable alterations of the trabecular architecture in addition to bone mineral density. The methodology applied is simple, widely available (computer with a film scanner) and less expensive and more informative.
